# A Novel Splice Donor Site Mutation Leading to Inherited Type I Protein S Deficiency

**DOI:** 10.3400/avd.cr.23-00076

**Published:** 2024-03-13

**Authors:** Yumi Sasaki, Jun Yamanouchi, Katsuto Takenaka

**Affiliations:** 1 Departments of Hematology, Clinical Immunology and Infectious Diseases, Ehime University Graduate School of Medicine, Toon, Ehime, Japan; 2 Division of Blood Transfusion and Cell Therapy, Ehime University Hospital, Toon, Ehime, Japan

**Keywords:** inherited protein S deficiency, *PROS1*, splice donor site mutation

## Abstract

Inherited Protein S (PS) deficiency is an autosomal dominant thrombotic disorder. We encountered a case of inherited type I PS deficiency following a close examination for recurrent pregnancy loss and identified the mutation responsible; a novel splice donor site mutation in intron 13 of the *PROS1* gene appeared to have caused a frameshift with premature termination at amino acid +551. These results will contribute to the creation of an accurate database and define the molecular basis for PS deficiency.

## Introduction

Protein S (PS) is a vitamin K-dependent anticoagulant protein produced mainly by the liver, vascular endothelial cells, and bone marrow megakaryocytes. It acts as a cofactor for activated protein C (PC) to degrade and inactivate activated coagulation factor V and factor VIII on platelets and vascular endothelial cells.^1)^ Congenital PS deficiency is an autosomal dominant disease, and thrombosis is often caused by factors that inactivate the coagulation system, such as surgery, trauma, pregnancy, and the use of oral contraceptive pills.^2)^ Aging also elevates the risk of venous thrombosis, with the odds of incidence being up to 8.5 times greater than in healthy individuals.^3)^ *PROS1*, which encodes PS, is located at position 11.2 on chromosome 3 and is a large 101-kb gene consisting of 16 introns and 15 exons. Congenital PS deficiency is caused by mutations in the *PROS1* gene. However, homozygous abnormalities are rare and most reported cases are caused by heterozygous abnormalities.^4^^,^^5)^

In the present study, we describe the case of a patient who was diagnosed with PS deficiency following a close examination for recurrent pregnancy loss (RPL). We performed direct sequencing and identified a novel mutation in *PROS1*; we therefore examined how the mutation reduced PS activity. We adopted the numbering standards of the Nomenclature Working Group, wherein the A in the ATG of the initiator Met codon was denoted as nucleotide +1 and the initial Met residue as amino acid +1.^6)^

## Case Report

The patient was a 36-year-old Japanese woman. Her blood test results showed reductions in PS activity (11%, normal range: 67%–164%), the total PS antigen level (57%, normal range: 65%–135%), and free PS antigen level (25%, normal range: 60%–150%). PS activity was measured by a clotting method, and PS antigen levels were measured by a latex agglutination test. Because PS activity and antigen levels decrease during pregnancy and the postpartum period,^7)^ her blood tests were performed 6 months after the miscarriage. She was retested 1 year later and had the same levels. Congenital PS deficiency can be classified into type I, in which both PS activity and total antigen levels are decreased; type II, in which the antigen level is normal but PS activity levels are decreased; and type III, in which both free PS antigen and PS activity levels are decreased. The patient was categorized into type I as both PS activity and total antigen levels were reduced. Although her father had no history of thrombosis, he was also diagnosed with type I PS deficiency as both PS activity and total antigen levels were reduced (PS activity: 17%, total PS antigen: 59%, free PS antigen: 25%). The patient had an antithrombin activity level of 94.4% (normal range: 70%–110%) and PC activity of 93.1% (normal range: 82%–112%). The patient tested negative for antiphospholipid antibodies (including anti-cardiolipin and anti-beta-2-glycoprotein I antibodies) and lupus anticoagulant.

To identify gene abnormalities in the *PROS1* gene, blood samples were collected from both the patient and her father, and DNA was extracted using the QIAamp DNA Blood Mini Kit (QIAGEN GmbH, Hilden, Germany). Primers were generated using extracted DNA as the template and subsequently placed in the intron region before and after each exon. Polymerase chain reaction (PCR) was performed for amplification, followed by direct sequencing to confirm the sequence. Lastly, base mutations were identified to determine their effects. The study was approved by the Human Genome and Genetic Analysis Research Ethics Committee at Ehime University School of Medicine (ethics committee approval number: 31-12), and was conducted after obtaining consent from patients.

**Figure 1:**
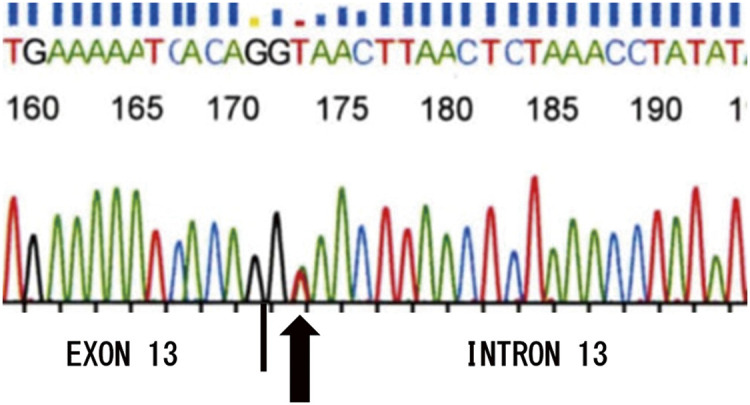
Fig. 1 Sequence of *PROS1* gene in the patient.

While there were no genetic mutations within the exon of the *PROS1* gene, we identified a heterozygous mutation from GT to GA in the intron two bases away from exon 13 (**Fig. 1**) (NM_000313.4:c.1985+2T>A). This heterozygous mutation from GT to GA does not satisfy the GT-AG rule, which is the most common type of intron; as such, the mutant allele failed to terminate the exons and the transcription of introns cannot be initiated. Our data also suggested that a stop codon was formed after the insertion of two new amino acids in exon 13. This mutation in exon 13 has never been reported in the literature and was also identified in the patient’s father. Thus, we hypothesized that this mutation contributed to the reduction of the PS level.

**Figure 2:**
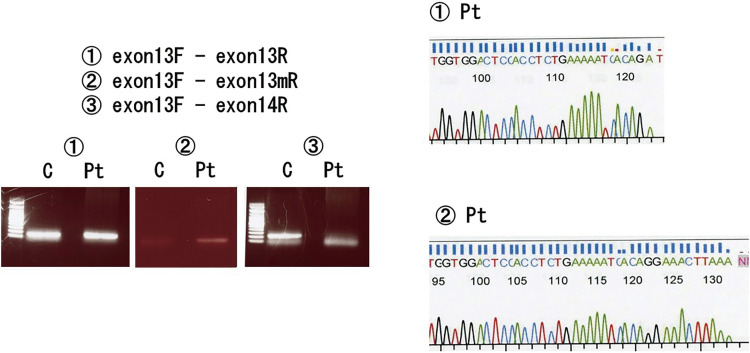
Fig. 2 RT-PCR assay of mRNA for *PROS1* gene. Three regions of the *PROS1* gene were amplified in a PCR reaction using the following primer pairs: the pair of experiment 1 was exon13F and exon13R coding exon 13, and the pair of experiment 2 was exon13F and exon13mR coding exon 13 mutant, and the pair of experiment 3 was exon13F and exon14R coding from exon 13 to exon 14. Amplified fragments were examined by sequence analysis. C: healthy control; Pt: patient; RT-PCR: reverse transcription polymerase chain reaction; PCR: polymerase chain reaction

To test this hypothesis, we extracted mRNA from the peripheral blood of the patient and generated primers to confirm the mutation site in exon 13. Total RNA was extracted from the blood samples using the RNeasy kit (QIAGEN). Three regions of the *PROS1* gene (exon 13, exon 13 mutant, and exon 13 to exon 14) were amplified in a PCR reaction using the following primer pairs: the first pair was a sense sequence, exon13F of 5′-gtatccagtgctgagggttg-3′ and its antisense sequence, exon13R of 5′-ggactccacctctgaaaaatcacag-3′ coding exon 13, and the second pair was exon13F and exon13mR of 5′-ggactccacctctgaaaaatcacaggaaacttaa-3′ coding exon 13 mutant, and the third pair was exon13F and exon14R of 5′-ggccctaagtctatgttccg-3′ coding from exon 13 to exon 14. Amplified fragments were examined by sequence analysis. We subsequently performed reverse transcription polymerase chain reaction (RT-PCR) of samples collected from the patient and healthy control. Four primers were placed within exons 13 and 14 to amplify mRNA by RT-PCR. Exon13F was placed upstream within exon 13, exon13R was placed downstream within exon 13, exon13mR was placed downstream within exon 13 containing the mutated site, and exon14R was placed upstream within exon 14. A nonspecific band was detected around 100 bp in all RT-PCRs and was also detected in PCRs in which only a primer set was added without mRNA, suggesting that this band might be a primer dimer. Results of RT-PCR using exon13F and exon13R are shown in Experiment 1. Expression of exon 13 was confirmed in both the patient and healthy control, as shown by the presence of the band. The expected amplification size is 147 bp. On the contrary, the band was only present in the patient sample when exon13F and exon13mR were used (Experiment 2). The expected amplification size is 156 bp. This indicates that mRNA was amplified by the exon13mR primer generated for exon 13 that contained the mutation. In other words, this showed that the mRNA of the patient contained the mutation. Using exon13F and exon14R, the band was only present in the healthy control sample and not in the patient sample (Experiment 3). Theoretically, the band is detected in the healthy control sample, while in the patient sample, the band is detected thinly because they are heterozygous for the normal and mutant allele. This case may also have a very thin band. The expected amplification size is 211 bp. This indicates that the mRNA translation ended at the stop codon in the patient; this was further confirmed in the RT-PCR product by direct sequencing (**Fig. 2**). Collectively, we demonstrated that the patient had a mutation from GT to GA in the intron two bases after exon 13 that prevented the termination of exon 13 and initiation of the intron, as well as the insertion of two new amino acids that led to the formation of the stop codon.

## Discussion

PS deficiency is a congenital thrombophilic predisposition that can lead to various thromboembolic diseases, including venous thromboembolism. Pregnancy, labor and delivery, and the postpartum period are risk factors for venous thromboembolism (VTE),^8)^ and PS deficiency may be diagnosed after VTE develops during pregnancy and the postpartum period. Several studies^9^^,^^10)^ have investigated the association between hereditary thrombophilia, including PS deficiency, and perinatal complications, but the results have been inconsistent. The perinatal impact of pregnancies complicated by PS deficiency remains unclear. On the other hand, it has been reported that miscarriages are significantly elevated in pregnancies complicated by PS deficiency.^11)^ PS deficiency may be diagnosed during a close examination for RPL, as in the present case.

We encountered a case of inherited type I PS deficiency following a close examination for RPL and identified the mutation responsible; a novel splice donor site mutation in intron 13 of the *PROS1* gene appeared to have caused a frameshift with premature termination at amino acid +551.

The study by Nakahara et al.^12)^ helps understand how the lack of exon 14 and beyond affects protein synthesis. The authors described a case in which a heterozygous change of AG to GG in an intron two bases before exon 14. As this mutation did not satisfy the GT-AG rule, the most common type of intron, the mutant allele failed to terminate the intron and initiate the transcription of exon 14. In the present case, the patient was diagnosed with type I congenital PS deficiency with PS activity of 10%, total PS antigen of 31%, and free PS antigen of 50%. These results suggest that deletion of exon 14 and beyond in PS results in type I congenital PS deficiency.

## Conclusion

A novel genetic mutation for congenital PS deficiency was identified in a patient by performing a detailed examination to determine the cause of RPL.

## Disclosure Statement

All authors have no conflict of interest.

## Author Contributions

Study conception: JY

Data collection: YS and JY

Analysis: JY

Investigation: YS and JY

Writing: YS and JY

Funding acquisition: KT

Critical review and revision: all authors

Final approval of the article: all authors

Accountability for all aspects of the work: all authors.
